# Response to: viva la VOSCE?

**DOI:** 10.1186/s12909-021-02941-z

**Published:** 2021-10-11

**Authors:** Lucinda Zahrah Motie, Shahil Kaini

**Affiliations:** University College London (UCL), Gower St, London, UK

## Abstract

Boyle et al. discuss the development and implementation of a Virtual Objective Structured Clinical Examination due to the COVID-19 pandemic lockdown precluding face-to-face Objective Structured Clinical Examinations, something we too as clinical medical students studying at University College London have experienced. We commend Boyle et al. for promptly creating and delivering this assessment. However, we believe this style of assessment has the potential to exacerbate the ethnic and social inequalities that currently exist within medical education. Going forward, it is imperative that the home environment is considered in an attempt to level the playing field.

Dear Editor,

We read with great interest the article by Boyle et al. [1] regarding the development and implementation of a Virtual Objective Structured Clinical Examination (VOSCE) during the COVID-19 pandemic lockdown. As clinical medical students studying at University College London (UCL), our summative Objective Structured Clinical Examinations (OSCE) was precluded due to the pandemic and so it was thought-provoking to read how the novel VOSCE successfully allowed the assessment of clinical performance.

We commend Boyle et al. [1] for their prompt ability to create and deliver a VOSCE. Although the main focus of the article was on delivery and logistical planning of this novel model, we believe there needs to be a greater discussion around how this style of assessment has the potential to exacerbate the ethnic and social inequalities that exist within medical education [2]. The environment in which a student takes part in virtual assessments can provide many challenges. There is a need for private, quiet space with adequate broadband. However, for a subset of students this may prove difficult. These individuals may include those from a background of a low socioeconomic status (SES), for whom housing characteristics are associated with SES [3]. Furthermore, some students may have additional responsibilities at home, such as being a carer or parent. International students may also struggle when living in a different time zone. Within literature, social differences have already been demonstrated to adversely impact medical student performance [2]. Going forward, it is imperative to consider the home environment of medical students when implementing virtual examinations in an attempt to level the playing field.

We understand that this format of examination was a temporary measure put in place due to the COVID-19 pandemic. If this style of assessment was to be utilised again, medical schools would have longer than Boyle et al. [1] to plan a VOSCE and so greater efforts could be made to minimise disparities. For example, at UCL, medical students who would have been disadvantaged by their home environment during the virtual single best answer summative were provided with an official space on campus. This was both quiet with reliable internet and adhered to social distancing requirements. Minority ethnic groups are already more likely to underperform and fail medical school examinations [4]. Following the COVID-19 pandemic, it is vital that any changes to the medical school curriculum do not further widen the attainment gap and disproportionately impact the socially disadvantaged.

## Data Availability

Not applicable.

## Abbreviations

VOSCE: Virtual Objective Structured Clinical Examination
OSCE: Objective Structured Clinical Examinations
SES: Socioeconomic status
